# Mortality, morbidity, and quality-of-life outcomes of patients requiring ≥ 14 days of mechanical ventilation: a 12-month post-intensive-care-unit cohort study

**DOI:** 10.5935/0103-507X.20190046

**Published:** 2019

**Authors:** Cláudia da Rocha Cabral, Cassiano Teixeira, Régis Goulart Rosa, Caroline Robinson, Daniel Sganzerla, Sérgio Henrique Loss, Priscila Lora, Vania Dezoti Micheletti

**Affiliations:** 1 Universidade Vale do Rio dos Sinos - São Leopoldo (RS), Brasil.; 2 Departamento de Terapia Intensiva, Hospital São Lucas, Pontifícia Universidade Católica do Rio Grande do Sul - Porto Alegre (RS), Brasil.; 3 Clínica Médica, Hospital Moinhos de Vento - Porto Alegre (RS), Brasil.; 4 Universidade Federal de Ciências da Saúde de Porto Alegre - Porto Alegre (RS), Brasil; 5 Departamento de Terapia Intensiva, Hospital de Clínicas de Porto Alegre - Porto Alegre (RS), Brasil.; 6 Departamento de Terapia Intensiva, Hospital Moinhos de Vento - Porto Alegre (RS), Brasil.; 7 Hospital Moinhos de Vento - Porto Alegre (RS), Brasil.

**Dear Editor,**

Intensive care unit (ICU) survivors suffer significant morbidity,^(1,2)^ and longer mechanical ventilation (MV)-dependency increases the probability that these subjects will suffer a "persistent inflammation-immunosuppression and catabolism syndrome".^(3-5)^ These patients have ongoing inflammation, manageable organ failure, ongoing protein catabolism, and poor nutrition, leading to cachexia; poor wound healing and immunosuppression with increased susceptibility to secondary infections; and poor long-term survival.^(3,4)^ These patients are classified as chronic critical illness (CCI) and present a protracted and complex ICU course that lasts for more than 7 days, they suffer from recurrent infections, organ dysfunction, malnutrition, weakness, cognitive decline, and prolonged institutionalization, and many fail to ever achieve functional independence and have poor long-term survival outcomes.^(6-8)^

The long-term prognosis of CCI patients (e.g., patients with MV for ≥14 days) was evaluated in the meta-analysis by Damuth et al.^(9)^ (39 studies from 16 countries), who reported a pooled mortality rate at hospital discharge of 29% (95% confidence interval - 95%CI, 26 - 32%), and 59% (95%CI, 56 - 62%) at 1 year. However, the quality of life (QoL) of these patients has not yet been described. Therefore, we developed a multicenter prospective cohort study involving 3 clinical-surgical ICUs (ICU 1: 31 beds, ICU 2: 10 beds, and ICU 3: 10 beds) during an 18-month period that enrolled 25 consecutive patients who were dependent on MV for ≥ 14 days, who were discharged from the ICU and who were followed-up for 12 months. The mean age of the patients was 63.4 ± 17.8 years, 48% of the patients were male, and the mean Charlson index score was 2.1 ± 1.9. The main cause of ICU admission was medical (68%), the median Acute Physiology and Chronic Health Disease Classification System (APACHE) II score was 17.7 [12.2 - 22.8], the mean number of days in the ICU was 17.7 [13.1 - 27.5] days, 92% of patients used vasopressor and inotropic drugs, 56% of patients needed continuous or intermittent dialysis, and 8% of patients used total parenteral nutrition during their ICU stay.

During a 12-month follow-up period, 36% of the patients died, 86% of patients needed an unplanned hospital readmission, and 11% (n = 1/11) of previous workers were able to return to work. Only 32% of patients were able to respond to self-administered questionnaires (Impact of Event Scale - IES [evaluation of Posttraumatic Stress Disorder - PTSD], HADS - Hospital Anxiety and Depression score [evaluation of depression and anxiety], and SF-12 (Short-Form of Health Survey Questionnaire [evaluation of quality of life]). Anxiety and depressive symptoms were present in 28.5% of patients, and no patient presented with PTSD. The score for the physical domain of SF-12 (45.5 ± 10.5) was lower than that for the mental domain (50.3 ± 10.4). Barthel's index was used to evaluate the capacity to carry out activities in daily living. The patients lost function in all 10 Barthel categories, but mainly in anal sphincter control (12.5% of patients, p = 0.01) and bladder sphincter control (25% of patients, p = 0.004) compared with the pre-ICU evaluation.

Only a small proportion of MV-dependent patients who survive to hospital discharge are discharged to their homes (19% [95%CI, 16 - 24%]).^(10)^ Nearly all patients with CCI leave the hospital with profound impairments in physical function, cognitive status, or both.^(10)^ Most of these patients require institutional care, and the frequency of hospital readmission during the first year after hospital discharge exceeds 40% of cases.^(11,12)^ Patients who are discharged to extended care facilities and cannot be sufficiently rehabilitated to return home within 6 months usually remain institutionalized until death.^(5,6,11,12)^ Some authors have demonstrated that fewer than 12% of CCI patients are alive and independent one year after their acute illness.^(13)^ In addition, prolonged dependency on ventilatory support can reduce life expectancy in the long term.^(13,14)^ Our patients presented low rates of anxiety, depression and PTSD, although a minority of the patients were able to answer the questionnaires. The probability of a return to employment was impressively low (11%), and a very high number of patients needed hospital readmission during follow-up (86% of the patients during the 12-month follow-up period). In contrast to these data, Euteneuer et al.^(12)^ evaluated 73 long-term survivors (>6 months) who had been transferred to a specialized weaning center due to prolonged MV (> 14 days) and weaning failure. They found that the presence of chronic respiratory failure (CRF) itself was the major determinant of QoL. Here, the underlying cause of CRF was the major factor that determined the degree of QoL impairment (measured by SF-36), with patients suffering from restrictive ventilatory disorders reporting the best QoL compared to patients with chronic obstructive pulmonary disease or neuromuscular diseases. Combes et al.^(10)^ evaluated the QoL (using the Nottingham Health Profile and St. George's Respiratory questionnaires) of 87 patients who were dependent on MV for ≥14 days after a median follow-up time of 3 years after ICU discharge. Compared to community-based controls, the patients had significantly more difficulties in all QoL domains except social isolation. The worst deficits were related to energy, sleep disorders and physical mobility. Two other studies measuring QoL in MV-dependent patients with tracheostomies^(15)^ and patients in a post-ICU facility^(16)^ also noted significant deficits in physical function and sleep, with generally good emotional health. As many as two-thirds of 6-month survivors from one ventilator weaning unit were unable to respond to cognitive assessments by telephone.^(17,18)^ This suggests that the overall QoL of most prolonged-MV cohorts is worse than what is able to be measured by QoL tools. Furthermore, the majority of these patients die within 12 months, and most of them experience severe symptoms in the process. Therefore, some authors^(4-6,19)^ suggest that palliative care should become a more prominent component of the management of prolonged-MV patients.

Our study has one important limitation, namely, its very small sample size (n = 25); however, the strength of the study is its complete evaluation of all the domains of the post-ICU critical syndrome. It presents a real-life report of the neuropsychiatric and physical characteristics of persistent critical disease, and the data demonstrate that 1/3 of ICU survivors who remain dependent on MV for more than 14 days die within 1 year of discharge. In addition, a small proportion of patients are able to return to employment, require many hospital readmissions, are largely unable to respond to self-reported QoL assessments and have anxiety, depression, and posttraumatic stress.

*Cláudia da Rocha Cabral Universidade Vale do Rio dos Sinos - São Leopoldo (RS), Brazil.* *Cassiano Teixeira Critical Care Department, Hospital São Lucas, Pontifícia Universidade Católica do Rio Grande do Sul - Porto Alegre (RS), Brazil; Internal Medicine, Hospital Moinhos de Vento - Porto Alegre (RS), Brazil; Universidade Federal de Ciências da Saúde de Porto Alegre - Porto Alegre (RS), Brazil and Critical Care Department, Hospital de Clínicas de Porto Alegre - Porto Alegre (RS), Brazil.* *Régis Goulart Rosa Critical Care Department, Hospital Moinhos de Vento - Porto Alegre (RS), Brazil* *Caroline Robinson Hospital Moinhos de Vento - Porto Alegre (RS), Brazil.* *Daniel Sganzerla Hospital Moinhos de Vento - Porto Alegre (RS), Brazil.* *Sérgio Henrique Loss Critical Care Department, Hospital de Clínicas de Porto Alegre - Porto Alegre (RS), Brasil.* *Priscila Lora Universidade Vale do Rio dos Sinos - São Leopoldo (RS), Brazil.* *Vania Dezoti Micheletti Universidade Vale do Rio dos Sinos - São Leopoldo (RS), Brazil.*
